# Quantitative Imaging of Changes in Astrocytic and Neuronal Adenosine Triphosphate Using Two Different Variants of ATeam

**DOI:** 10.3389/fncel.2020.00080

**Published:** 2020-04-21

**Authors:** Rodrigo Lerchundi, Na Huang, Christine R. Rose

**Affiliations:** Institute of Neurobiology, Faculty of Mathematics and Natural Sciences, Heinrich Heine University Düsseldorf, Düsseldorf, Germany

**Keywords:** adenosine triphosphate, FRET imaging, ATeam, neuro-metabolic coupling, astrocyte

## Abstract

Genetically encoded nanosensors such as the FRET-based adenosine triphosphate (ATP) sensor ATeam enable the measurement of changes in ATP levels inside cells, promoting our understanding of metabolic interactions between astrocytes and neurons. The sensors are usually well characterized *in vitro* but display altered properties when expressed inside cells, precluding a meaningful conversion of changes in FRET ratios into changes in intracellular ATP concentrations ([ATP]) on the basis of their *in vitro* properties. Here, we present an experimental strategy for the intracellular calibration of two different variants of ATeam in organotypic tissue slice culture of the mouse brain. After cell-type-specific expression of the sensors in astrocytes or neurons, slices were first perfused with a saline containing the saponin β-escin to permeabilize plasma membranes for ATP. Next, cells were depleted of ATP by perfusion with ATP-free saline containing metabolic inhibitors. Finally, ATP was re-added at defined concentrations and resulting changes in the FRET ratio recorded. When employing this protocol, ATeam1.03 expressed in astrocytes reliably responds to changes in [ATP], exhibiting an apparent *K*_D_ of 9.4 mM. The high-affinity sensor ATeam1.03^YEMK^ displayed a significantly lower intracellular *K*_D_ of 2.7 mM. On the basis of these calibrations, we found that induction of a recurrent neuronal network activity resulted in an initial transient increase in astrocytic [ATP] by ~0.12 mM as detected by ATeam1.03^YEMK^, a result confirmed using ATeam1.03. In neurons, in contrast, [ATP] immediately started to decline upon initiation of a network activity, amounting to a decrease by an average of 0.29 mM after 2 min. Taken together, our results demonstrate that ATeam1.03^YEMK^ and ATeam1.03 display a significant increase in their apparent *K*_D_ when expressed inside cells as compared with *in vitro*. Moreover, they show that both ATeam variants enable the quantitative detection of changes of astrocytic and neuronal [ATP] in the physiological range. ATeam1.03^YEMK^, however, seems preferable because its *K*_D_ is close to baseline ATP levels. Finally, our data support the idea that synchronized neuronal activity initially stimulates the generation of ATP in astrocytes, presumably through increased glycolysis, whereas ATP levels in neurons decline.

## Introduction

Adenosine triphosphate (ATP) is the basic intracellular energy currency of living cells (Plattner and Verkhratsky, 2016). In the brain, most of the ATP is used by ion pumps expressed at the plasma membrane and in membranes of intracellular organelles. The latter include the vesicular ATPase (v-ATPase), which is responsible for the acidification of presynaptic vesicles that drives the uptake of neurotransmitters like glutamate (Cotter et al., 2015). At the plasma membrane, the Ca^2+^-ATPase pumps Ca^2+^ out of the cell and thereby contributes to the maintenance of a low intracellular Ca^2+^ and the recovery from intracellular Ca^2+^ transients (Guerini et al., 2005). One of the major consumers of ATP in mammalian tissue is the plasma membrane Na^+^/K^+^-ATPase (NKA; Erecińska and Silver, 1994). It generates the transmembrane Na^+^ and K^+^ gradients, transporting three Na^+^ out of the cell while importing two K^+^, and it hydrolyzes ATP to ADP and inorganic phosphate during each transport cycle (Skou and Esmann, 1992; Kaplan, 2002). Although it is ubiquitously expressed, its activity is particularly critical for brain function (Somjen, 2004).

At the cellular level, neurons and astrocytes in fact expend most of their ATP on maintaining a low intracellular Na^+^ concentration ([Na^+^]_i_) and to counteract Na^+^ influx brought about by excitatory activity and Na^+^-dependent secondary-active transport (Erecińska and Silver, 1994; Harris et al., 2012; Gerkau et al., 2017). Metabolic pathways for ATP generation to support the NKA, however, differ between both. Neurons exhibit a high rate of ATP consumption but mostly rely on glucose and lactate to drive glycolysis and the tricarboxylic acid (TCA) cycle, because they lack glycogen (Bak et al., 2018). Astrocytes, on the other hand, consume less ATP (Harris et al., 2012) but contain large intracellular reservoirs of glycogen, which can be broken down to generate glucose-1-phosphate to be fed into glycolysis (Brown and Ransom, 2007; Bak et al., 2018).

The metabolic response to a neuronal activity also seems to differ between both cell types. Different approaches to monitor cellular ATP have suggested that following application of glutamate or induction of electrical activity, neurons experience a decline in intracellular ATP levels (Ainscow et al., 2002; Mollajew et al., 2013; Trevisiol et al., 2017; Gerkau et al., 2019). In astrocytes, but not in neurons, increasing extracellular K^+^ to mimic excitatory neuronal activity results in an increase in ATP levels, consistent with an increase in astrocyte glycolysis (Fernández-Moncada et al., 2018; Lerchundi et al., 2019a). Notably, glycogen breakdown in astrocytes can be elicited by cAMP and Ca^2+^ and has been implicated in learning and memory (Gibbs et al., 2006; Duran et al., 2013; Alberini et al., 2018; Bak et al., 2018). These observations suggest a direct relation between astrocyte metabolism and neuronal performance, which might be mediated through a transfer of glycolytically derived lactate from astrocytes to neurons. Although there is a lot of evidence for this so-called astrocyte–neuron lactate shuttle (ANLS; Pellerin and Magistretti, 2012; Mächler et al., 2016; Barros and Weber, 2018), there are also opposing views (Dienel, 2014, 2019; Bak and Walls, 2018).

To address such questions and study neuro-metabolic coupling, the detection of metabolites and of changes thereof during different manipulations in neurons and astrocytes is of great interest. In the last decade, imaging with genetically encoded nanosensors was introduced, enabling the dynamic measurement of metabolites such as ATP in living cells (e.g., Imamura et al., 2009; Tantama et al., 2013). However, although these sensors can reliably be calibrated *in vitro* (in cell-free conditions), their properties usually change in the intracellular environment. This, for example, includes a change in *K*_D_ (Yaginuma et al., 2014) or in their temperature sensitivity (e.g., Lerchundi et al., 2019a). A quantitative measurement of ATP concentrations (ATP) has therefore been particularly challenging under *in situ* conditions (i.e., in the intact tissue), and most studies performed so far report only relative changes in ATP levels inside cells.

In the present study, we have developed a new strategy to selectively permeabilize neurons and astrocytes in organotypic brain slice cultures to enable the free exchange of ATP across the plasma membrane. We show that this procedure allows a full calibration of the genetically encoded, FRET-based nanosensor ATeam inside the cells, enabling the quantitative measurement of changes in intracellular [ATP] in the intact tissue. Furthermore, the results presented in this work support the idea that neuronal activity differentially affects [ATP] in neurons and astrocytes.

## Materials and Methods

### Ethics Statement

This study was carried out in strict accordance with the institutional guidelines of the Heinrich Heine University Düsseldorf as well as the European Community Council Directive (2010/63/EU). Following the guidelines of the European Commission (Close et al., 1997), animals up to 10 days old were quickly decapitated. All experiments involving brain slices were approved by the Animal Welfare Office at the Animal Care and Use Facility of the Heinrich Heine University Düsseldorf (Institutional Act number O50/05).

### Preparation of Organotypic Brain Slice Cultures

Organotypic brain tissue slice cultures were prepared following a protocol described recently (Schreiner et al., 2013; Lerchundi et al., 2019a,b) that was adapted from Stoppini et al. (1991). Briefly, mice (both sexes) of postnatal days 6–8 (P6–P8) were quickly decapitated, and brains were immediately placed in ice-cold saline containing (in mM): 125 NaCl, 2.5 KCl, 2 CaCl_2_, 1 MgCl_2_, 1.25 NaH_2_PO_4_, and 26 NaHCO_3_ that was carbogenated (5% CO_2_/95% O_2_), resulting in a pH of 7.35–7.4. Next, brains were separated into hemispheres and cut into 250-μm-thick parasagittal slices using a vibrating blade microtome (HM 650 V; Thermo Fisher Scientific, Waltham, MA, USA).

Hippocampus and adjacent cortex were isolated under semi-sterile conditions, and slices were washed five times with acidified Hanks solution (Sigma–Aldrich, Munich, Germany), after which they were immediately transferred onto Biopore membranes (0.4-μm pore size Millicell standing insert; Merck Millipore, Burlington, MA, USA). Slices were kept in an incubator at 36.5°C between 10 and 21 days at an interface between humidified carbogen (95% O_2_/5% CO_2_) and culture medium (Gee et al., 2017) containing minimum essential medium (MEM; M7278; Sigma–Aldrich, Munich, Germany), 20% heat-inactivated horse serum (Origin: Brazil; Thermo Fisher Scientific, Waltham, MA, USA), 1 mM L-glutamine, 0.01 mg/ml insulin, 14.5 mM NaCl, 2 mM MgSO_4_, 1.44 mM CaCl_2_, 0.00125% ascorbic acid, and 13 mM of D-glucose. The medium was replaced every 3 days.

### Transduction of Organotypic Slices

Two variants of the genetically encoded nanosensor ATeam were employed: ATeam1.03^YEMK^ and ATeam1.03 (Imamura et al., 2009). Transduction was performed as described in detail recently (Lerchundi et al., 2019b). For transduction of astrocytes, a dilution of 0.5 μl of an adeno-associated vector (AAV) carrying the code for ATeam under the astrocyte-specific promoter glial fibrillary acidic protein (GFAP; AAV5/2 ATeam1.03^YEMK^, titer: 2.16 × 10^12^ viral genome/ml or AAV5/2 ATeam1.03, titer: 1.43 × 10^12^ viral genome/ml) were directly applied to the top of each slice at 1–3 days *in vitro* (DIV). For specific transduction of neurons, the promoter human synapsin1 (hSyn1) was employed (AAV9/2 ATeam1.03^YEMK^, titer: 1.38 × 10^12^ viral genome/ml). Transduced slices were placed back into the incubator and maintained at 36.5°C (95% O_2_/5% CO_2_) for at least six more days until the experiments were performed.

### FRET-Based ATP Imaging

Experiments in organotypic cultured slices were performed at room temperature (20–22°C) in an experimental bath that was constantly perfused with saline at 2–2.5 ml/min. Unless otherwise stated, the standard saline was composed of (in mM) the following: 136 NaCl, 3 KCl, 2 CaCl_2_, 1 MgCl_2_, 1.25 NaH_2_PO_4_, 24 NaHCO_3_, and 5 glucose, bubbled with 95% O_2_/5% CO_2_ to obtain a pH of between 7.35 and 7.4. ATP imaging was performed using a wide-field fluorescence microscope (Nikon Eclipse FN-I; Nikon GmbH Europe, Düsseldorf, Germany) equipped with an Achroplan 40× objective (water immersion, N.A. 0.8; Zeiss, Göttingen, Germany).

Slices were excited at 435 nm using a Poly-V ultrafast switching monochromator (Thermo Fisher Scientific/FEI; Planegg, Germany). Resulting fluorescence emission from eCFP (donor; emission peak at 475 nm) and Venus (acceptor; emission peak at 527 nm) was split at 500 nm with an image splitter (A12801-01; Hamamatsu Photonics GmbH, Herrsching, Germany) and filtered with band pass filters at 483/32 and 542/27, respectively (AHF Analysentechnik AG, Tübingen, Germany). For image collection, a CMOS camera (Orca 4 LT Plus; Hamamatsu) was used. Fluorescence was analyzed from regions of interest (ROIs) representing cell somata, employing the NIS-Elements software (Nikon). After background correction, the FRET ratio (Venus/eCFP) was calculated and analyzed using OriginPro 9 Software (OriginLab Corporation, Northampton, MA, USA).

### Saline for Calibration of ATeam

For calibration of ATeam, organotypic brain slices were placed in an experimental chamber and perfused with HEPES-based saline (in mM: 125 NaCl, 2.5 KCl, 2 CaCl_2_, 2 MgCl_2_, 1.25 NaH_2_PO_4_, and 25 HEPES, pH 7.4). Slices were then exposed to a permeabilization saline mimicking intracellular conditions, containing (in mM) the following: 145 KMeSO_3_, 40 KCl, 12.5 HEPES, 10 NaCl, 5 Mg-ATP, and 0.5 Na_3_-GTP, pH 7.3. In addition, 30 μM β-escin was added to permeabilize the plasma membrane. Next, slices were perfused with a calibration saline containing (in mM) the following: 25 NaCl, 129 K-gluconate, 10 HEPES and 10 MgCl_2_, 5 NaN_3_, 2-deoxy-D-glucose (2-DG), adjusted to pH 7.3 with KOH, to deplete cells of ATP and inhibit cellular ATP generation. In some experiments, the calibration saline also contained 0.1 mM sodium orthovanadate (Na_3_VO_4_). Finally, ATP was re-added at different concentrations, ranging from 1 to 10 mM, and changes in the FRET ratio were recorded.

### Confocal Imaging

Images for documentation of plasma membrane permeabilization were taken at a confocal laser scanning microscope (Eclipse C1; Nikon Microscope Solutions, Düsseldorf, Germany) equipped with a water immersion objective (Fluor 60×/0.80 W DIC M ∞/0 WD 2.0; Nikon). ATeam was excited using a 488-nm wavelength argon laser, and emission was collected at >515 nm. Three-dimensional (3D) projections were constructed from z-stacks (step size 1.05 μm) using NIS-Elements software (Nikon).

### Data Analysis and Presentation

FRET-based ATP imaging data were analyzed using Windows Excel and Origin Pro software (OriginLab Corporation, Northampton, MA, USA). Data are presented as means ± SD (standard deviation) and illustrated as box and whisker plots, indicating median (line), mean (dot), interquartile range (IQR; box), and SD (whiskers). Statistical analysis was performed with either a *t*-test or a one-way ANOVA followed by *post hoc* Bonferroni test. “*n*” indicates number of cells and “*N*” number of individual experiments; ***:*p* < 0.001. Each set of experiments was performed on tissue slices prepared from at least three different animals.

## Results

### Permeabilization Protocol

The aim of this work was to establish a quantitative measurement of changes in intracellular ATP concentration ([ATP]) in organotypic tissue slices of the mouse hippocampus and cortex and to compare the suitability of two different variants of the FRET-based nanosensor ATeam. To this end, we first developed a procedure for the calibration of the high-affinity variant ATeam1.03^YEMK^ and the lower-affinity probe ATeam1.03 in astrocytes following a strategy recently introduced for neurons (Gerkau et al., 2019). Such calibration *in situ* requires: (a) a cell-type-specific expression of the sensor in astrocytes; (b) a free, unrestricted passage of ATP across cellular plasma membranes; and (c) a nominal depletion of cells from ATP to obtain a reliable reference and normalization point (0 ATP).

For cell-type-specific expression of the sensor, organotypic brain slices were transduced with an AAV carrying the code for ATeam1.03^YEMK^ under the control of the GFAP promoter, resulting in ATeam1.03^YEMK^ expression in astrocytes only as reported before (Lerchundi et al., 2019a; see also Figure 2). Transfected slices were transferred to an experimental chamber and perfused with HEPES-buffered saline, and regions with astrocytes expressing the sensor were identified based on fluorescence emission generated by the donor and/or acceptor (see also Figure 2). Most recordings were performed in astrocytes located in the *stratum oriens* of the hippocampal CA1–3 regions or in cortical layers II and III.

**Figure 1:**
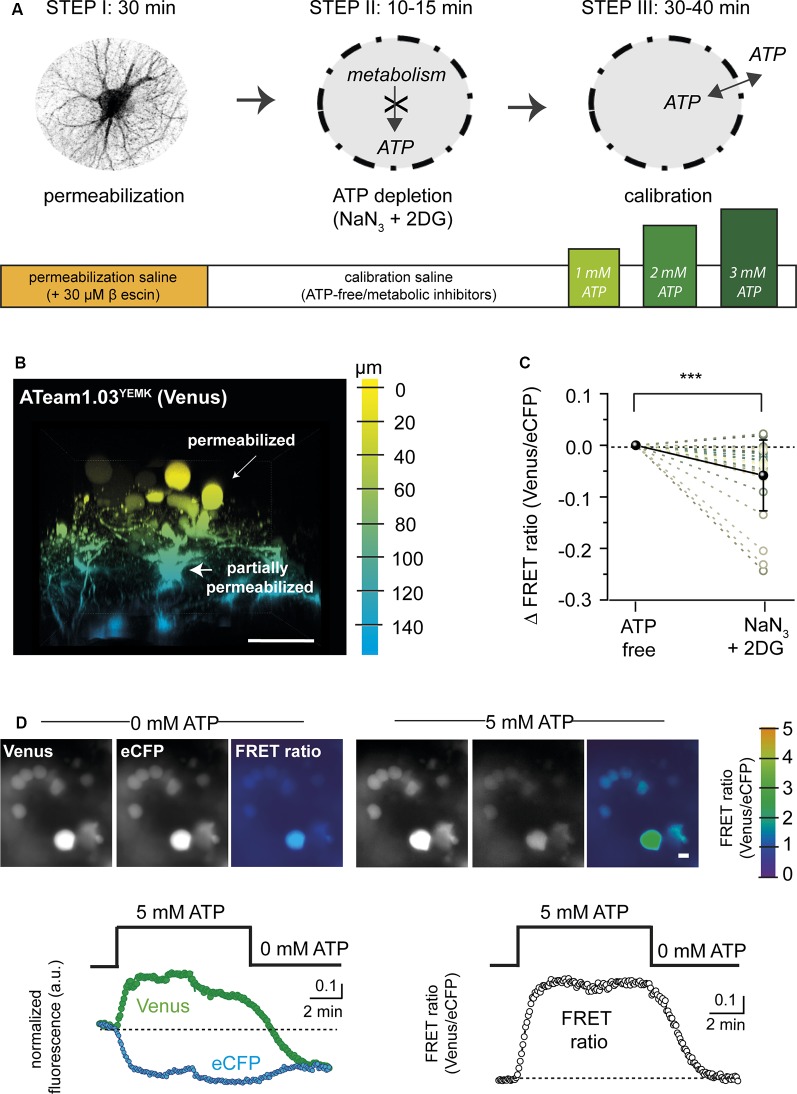
Permeabilization protocol for *in situ* calibration of ATeam. **(A)** Schematic summary showing the different steps (I–III) and the approximate time required for permeabilization (Step I), adenosine triphosphate (ATP) depletion (Step II), and calibration (Step III) of ATeam in organotypic tissue slice culture.** (B)** Three-dimensional (3D) reconstruction of an organotypic cultured brain slice (cortex) expressing ATeam1.03^YEMK^ in astrocytes after 30 min of incubation with 30 μM of β-escin (see Step I in **A**). The color code indicates the depth, with 0 μm representing the slice surface. Scale bar: 50 μm. Note that cell bodies on the surface (yellow) are swollen and rounded, indicating a successful permeabilization of the plasma membrane. Cells in deeper layers (blue) are only slightly swollen, pointing towards an incomplete permeabilization.** (C)** Change in the FRET ratio of permeabilized cells in ATP-free saline before (ATP-free) and after exposure to 5 mM of NaN_3_ and 2 mM of 2-deoxy-D-glucose (2-DG). Note that additional inhibition of cellular ATP production results in a further decrease in the ATeam FRET ratio (***p < 0.001; *n* = 27; *N* = 3). **(D)** Top: images of Venus and eCFP fluorescence and the corresponding FRET ratio (color coded, scale on the very right) in fully permeabilized cells in ATP-free saline (left) and after addition of 5 mM of ATP (right). Scale bar: 10 μm. Bottom left: traces showing changes in the Venus fluorescence (green dots) and in the eCFP fluorescence (cyan dots) in fully permeabilized cells when switching from ATP-free conditions to 5 mM of ATP. Right: corresponding changes in the FRET ratio.

**Figure 2:**
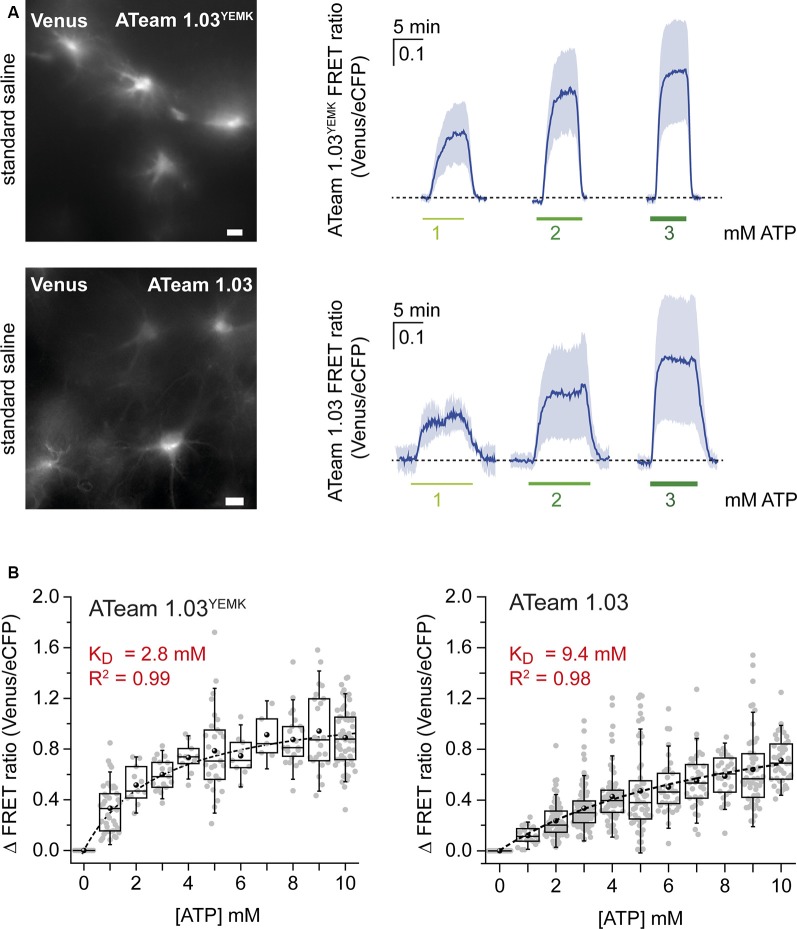
Calibration of ATeam1.03^YEMK^ and ATeam1.03 in astrocytes. **(A)** Left: images of the Venus fluorescence of astrocytes in organotypic brain slices expressing either ATeam1.03^YEMK^ (top) or ATeam1.03 (bottom). Scale bars: 10 μm. Right: changes in the FRET ratio of ATeam1.03^YEMK^ (top) and ATeam1.03 (bottom) in fully permeabilized cells after switching from ATP-free saline to 1, 2, and 3 mM of ATP and back. **(B)** Box plots showing the FRET ratio of ATeam1.03^YEMK^ (left) and ATeam1.03 (right) at different ATP concentrations, normalized to 0 mM of ATP. The dotted black lines represent Michaelis–Menten fits.

For permeabilization, we first employed the steroidal saponin digitonin to promote the formation of pores in the plasma membrane and to allow an unrestricted exchange of ATP between the extracellular space and the cytosol. Incubation of organotypic slices with 12 μM of digitonin, however, resulted in a rapid decrease in the emission fluorescence of the FRET pair (*n* = 35, *N* = 3). Emission was almost completely lost after 25 min of incubation, suggesting the leakage of the sensor from the cells by a destabilization of the plasma membrane (Sudji et al., 2015).

As an alternative, we then used the saponin β-escin (Figure 1, Step I). Slices expressing the sensor were perfused with a permeabilization saline containing 30 μM β-escin. In addition, the ion composition of the permeabilization saline was adapted to better reflect intracellular concentrations (e.g., high K^+^ and low Na^+^, as detailed in the “Materials and Methods” section). About 30 min after switching to the permeabilization saline, cells in the uppermost tissue layers rounded up and showed swollen cell bodies, indicative of a successful permeabilization (Figures 1B,D). The majority of these astrocytes most likely represented cells of the glial scar covering the organotypic tissue slice culture (Lerchundi et al., 2019b). Astrocytes in deeper tissue layers, in contrast, still partially maintained their morphology and ramification, pointing towards an only partial permeabilization of their cell membranes (Figure 1B).

In the next step, the tissue was depleted of ATP. To this end, slices were exposed to a calibration saline nominally free of ATP, containing NaN_3_ (5 mM) to block mitochondrial respiration and 2-DG (2 mM) to block glycolysis for 10–15 min (Figure 1, Step II). The metabolic inhibitors were introduced because we found that their application resulted in a further decrease in the FRET ratio of rounded (fully permeabilized) cells (by 0.06 ± 0.07; *n* = 27, *N* = 3) as compared with ATP-free saline, suggesting a nominal depletion of intracellular ATP only under these conditions (Figure 1C).

In the last step (Figure 1, Step III), the FRET ratio was recorded from fully permeabilized cells in upper tissue layers, and the baseline was determined in ATP-depleted conditions (Figure 1D). ATP was then re-added to the saline and resulting changes in the FRET ratio recorded. Figure 1D illustrates that a change from 0 to 5 mM of ATP and back induced a reversible change in the emission of the FRET pair (*n* = 29, *N* = 6), with an increase in Venus emission (Figure 1D left, green trace) and an opposite effect on eCFP emission (Figure 1D left, cyan trace). While fluorescence emission from both the acceptor and the donor was subject to an overall decline (most likely due to bleaching), calculating the FRET ratio resulted in a stable signal, reporting an average increase of the FRET ratio by 0.79 ± 0.32 when switching from 0 to 5 mM of ATP and a return to the initial baseline when switching back to 0 mM of ATP (Figure 1D, right).

### Full Calibration of ATeam1.0^YEMK^ and ATeam1.03 in Astrocytes

In cuvette calibrations, ATeam1.03^YEMK^ exhibits a *K*_D_ of ~0.2 mM at room temperature (Imamura et al., 2009). Baseline [ATP] in neurons and astrocytes is around 2 mM (Fukuda et al., 1983; Ainscow et al., 2002; Mollajew et al., 2013; Rangaraju et al., 2014; Toloe et al., 2014; Pathak et al., 2015), and the low *in vitro K*_D_ of ATeam1.03^YEMK^ suggests that it might not be optimally suited for detection of changes in intracellular [ATP] at room temperature. We therefore employed the strategy described above to perform a full calibration of ATeam1.03^YEMK^ in astrocytes and to determine its apparent *K*_D_ *in situ*.

For *in situ* calibration, organotypic slices expressing ATeam1.03^YEMK^ in astrocytes (Figure 2) were subjected to β-escin and depleted of ATP in the presence of metabolic inhibitors. In addition to NaN_3_ and 2-DG, sodium orthovanadate (Na_3_VO_4_; 100 μM), a non-selective ATPase inhibitor, was applied in this set of experiments. Exposing the slices to 1, 2, and 3 mM of ATP resulted in changes in the cellular FRET ratio of 0.33 ± 0.19, 0.52 ± 0.15, and 0.6 ± 0.13, respectively (*n* = 43, *N* = 3; Figure 2, upper panel). Full calibration for [ATP] between 0 and 10 mM showed a saturation behavior in this concentration range (*n* ≥ 5, *N* ≥ 3; Figure 2B). Fitting the data with a Michaelis–Menten fit revealed an apparent *K*_D_ value of 2.8 mM for ATeam1.03^YEMK^ in astrocytes (Figure 2B).

The same procedure was applied for the ATeam variant 1.03 that exhibits an *in vitro* *K*_D_ of below 1 mM at room temperature (Imamura et al., 2009). Again, when the sensor was expressed in astrocytes, it responded with increases in the FRET ratio upon exposure to increases in [ATP]. Changing from ATP-free condition to 1, 2, and 3 mM of ATP resulted in changes in the FRET ratio of 0.12 ± 0.07, 0.23 ± 0.14, and 0.43 ± 0.21, respectively (*n* = 125, *N* = 5; Figure 2, lower panel). Fitting the data obtained for the concentration range between 0 and 10 mM of ATP (*n* ≥ 21, *N* ≥ 3) by a Michaelis–Menten fit revealed an apparent *K*_D_ of 9.4 mM for ATeam1.03 (Figure 2B, right).

Taken together, these results demonstrate that the sensitivity of the two ATeam variants to ATP follows a sigmoidal behavior. Inside cells, like *in vitro*, the apparent affinity of ATeam1.03 for ATP is significantly lower than that of ATeam1.03^YEMK^. Notably, however, the *K*_D_’s of both sensors as determined *in situ* are significantly higher than those *in vitro*.

### ATP Sensitivity of ATeam1.03^YEMK^ Expressed in Neurons

**Figure 3:**
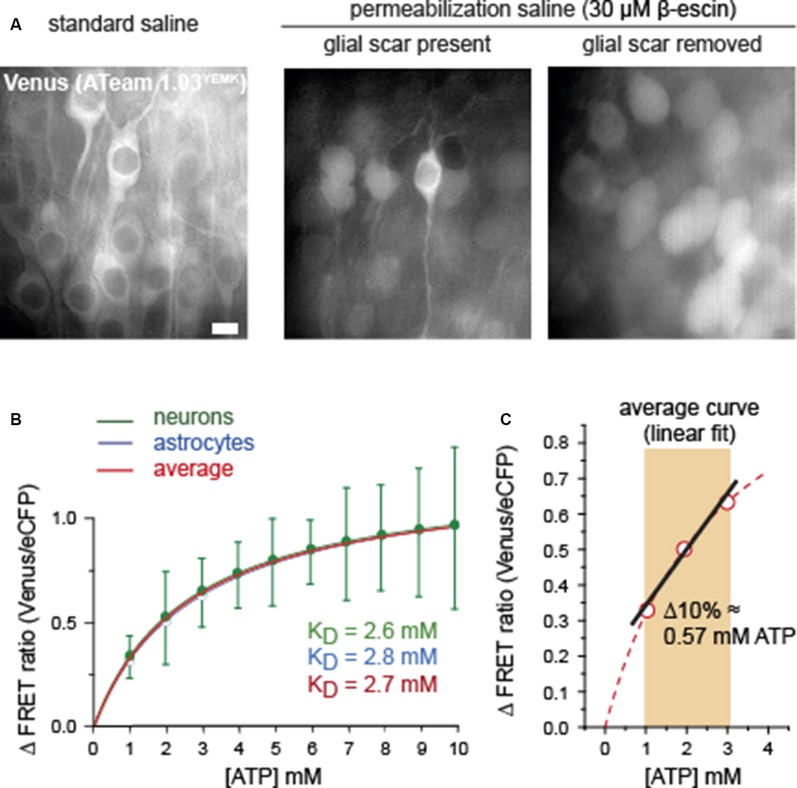
Comparison of calibration of ATeam1.03^YEMK^ in neurons and astrocytes. **(A)** Images of the Venus fluorescence of neurons in organotypic brain slices expressing ATeam1.03^YEMK^ in physiological saline (left), and after exposure to β-escin with the glial scar present (center) and absent (right). Note that full permeabilization, as indicated by swollen and round cell bodies, is only obtained after removal of the glial scar. Scale bar: 10 μm.** (B)** Mean values and Michaelis–Menten fits of the FRET ratio of ATeam1.03^YEMK^ at different ATP concentrations, normalized to 0 mM of ATP. Green, neurons (±SD); blue, astrocytes (same data and SD as shown in Figure 2B). The red curve shows the average of both. **(C)** Linear fit of the averaged calibration curve obtained from **(B)** between 1 and 3 mM of ATP.

We next compared the ATP sensitivity of ATeam1.03^YEMK^ as determined in astrocytes with that of the same sensor when expressed in neurons. To this end, organotypic slices were transduced with an AAV vector hosting the hSyn1 promoter, resulting in a specific neuronal expression of ATeam1.0^YEMK^ (Figure 3; Gerkau et al., 2019). As observed for astrocytes, incubation with a saline containing β-escin to permeabilize plasma membranes for ATP (see Figure 1, Step II) resulted in a swelling of cells in the upper tissue layers (Figure 3). Still, the swelling of neuronal cell bodies was less pronounced than that of astrocytes observed before (*N* = 5; compare Figures 1B,D). However, when opening the glial scar covering organotypic slices as recently described (Lerchundi et al., 2019b), neurons rounded up more strongly in response to perfusion with β-escin, suggesting a better permeabilization of the cells (*N* = 5; Figure 3).

Full calibration of ATeam1.03^YEMK^ in neurons by exposing permeabilized slice preparations to different [ATP] between 0 and 10 mM (*n* = 34, *N* = 5) revealed a nearly identical Michaelis–Menten relationship than that of astrocytes (Figure 3B). Specifically, the apparent *K*_D_ determined for neurons was 2.6 mM, as compared with 2.8 mM obtained for glial cells (Figure 3B). Because of their comparable behavior, an average calibration curve was established to represent a standard, applicable for both cell types, resulting in a *K*_D_ of 2.7 mM (Figure 3B, red trace).

As described above and evident from Figures 1B,D, 3A, the permeabilization of the tissue slices with β-escin resulted in strong cellular swelling. In addition, cell bodies moved considerably, requiring repeated re-focusing of the preparation. The repeated optical re-adjustment during permeabilization inherited artificial shifts in the ATeam FRET ratio. The latter impeded a reliable comparison of cellular baseline FRET ratios in standard saline with the FRET ratios in permeabilized, ATP-free conditions, precluding a proper and unbiased determination of baseline cellular [ATP]. Based on earlier studies, reporting a baseline [ATP] of ~2 mM in neurons and astrocytes (see above), we applied a linear fit to the standard calibration curve of ATeam1.03^YEMK^ between 1 and 3 mM (Figure 3C). A 10% change in the FRET ratio thereby translated into a change of [ATP] by 0.57 mM (Figure 3C). Linear fitting of the ATeam1.03 calibration curve obtained for astrocytes between 1 and 3 mM of ATP yielded a change in ATP by 0.93 mM for a 10% change in the FRET ratio (not illustrated).

In summary, these results indicate that ATeam1.03^YEMK^ exhibits a similar ATP sensitivity when expressed in neurons or in astrocytes of organotypic slice cultures of the mouse brain. Linearization of the calibration curve between 1 and 3 mM of ATP enables a direct quantitative determination of changes in intracellular [ATP] in this range based on changes in the ATeam FRET ratio.

### Changes in Cellular Adenosine Triphosphate Induced by Neuronal Network Activity

To study the effect of a neuronal activity on [ATP] in astrocytes and neurons, organotypic tissue slices expressing ATeam1.03^YEMK^ either in astrocytes or in neurons were exposed to a saline containing 10 μM of bicuculline methiodide, a competitive antagonist of GABA_A_ receptors. In addition, the Mg^2+^ concentration of the saline was reduced from 1 to 0.5 mM to reduce its voltage-dependent block of NMDA receptors. Such a disinhibition induces regular burst firing of the entire neuronal network, accompanied by a transient Na^+^ loading of both neurons and astrocytes in hippocampal slice preparations (Karus et al., 2015; Gerkau et al., 2019).

**Figure 4:**
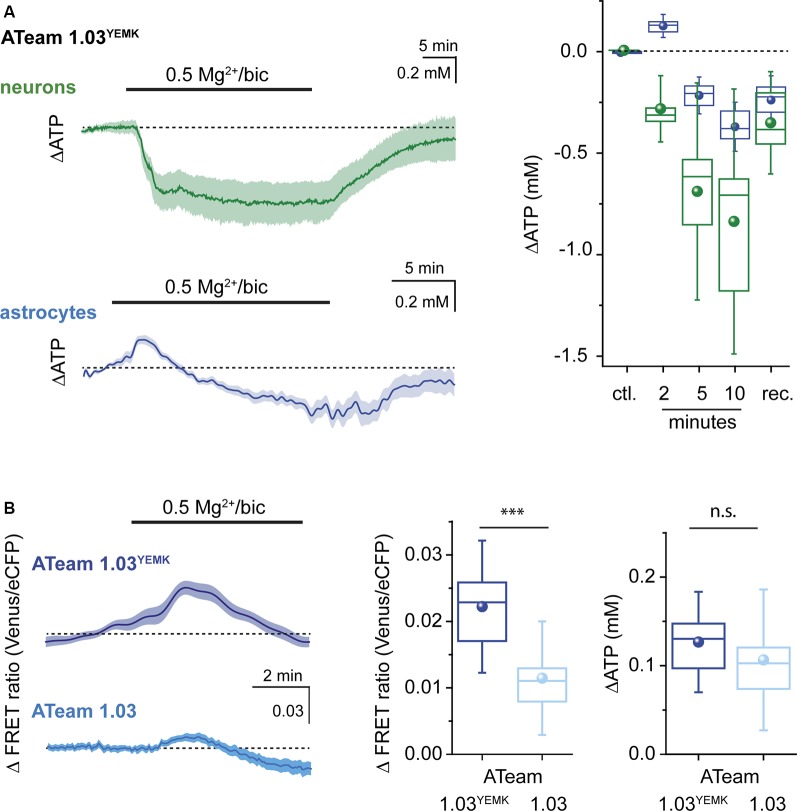
Changes in ATP evoked by recurrent network activity. **(A)** Left: changes in the [ATP] in neurons (green) and astrocytes (blue) as reported by ATeam1.03^YEMK^ in organotypic brain slice culture following induction of recurrent network activity induced by exposure to bicuculline (bic) and low extracellular Mg^2+^ as indicated by the bar. Shown are traces from individual cell bodies (light color) as well as the averages thereof (dark traces). Right: box plots illustrating the peak changes in the [ATP] in neurons (green) and astrocytes (blue) in the control (ctl.), 2, 5, and 10 min after induction of recurrent network activity and after wash-out (rec.). **(B)** Left: changes in the FRET ratio of astrocytes, expressing ATeam1.03^YEMK^ (top) or ATeam1.03 (bottom) after induction of recurrent network activity. Shown are traces from individual cell bodies (light color) as well as the averages thereof (dark traces). Center: box plot illustrating the peak increase in the FRET ratio in astrocytes expressing ATeam1.03^YEMK^ (dark blue) or ATeam1.03 (light blue) 2 min after induction of recurrent network activity. Right: box plot illustrating the peak increase in the [ATP] of astrocytes expressing ATeam1.03^YEMK^ (dark blue) or ATeam1.03 (light blue). ***p< 0.001; n.s.: not significant.

When switching from standard saline to saline with bicuculline and reduced Mg^2+^, [ATP] in neurons rapidly decreased by on average 0.29 ± 0.1 mM (*n* = 60, *N* = 3) as determined after 2 min of exposure (Figure 4). Neuronal [ATP] continued to decrease, reaching 0.69 ± 0.35 mM below the initial baseline after 5 min and 0.85 ± 0.44 after 10 min (Figure 4). Of note, albeit [ATP] dropped in each neuron investigated, there was a rather large variability in the magnitude of the neuronal response (Figure 4). After re-perfusion with standard saline, neuronal [ATP] slowly recovered towards initial baseline, which was, however, not fully reached within the observation period of 25 min.

In contrast to neurons, astrocytes responded to the induction of a recurrent network activity by transiently increasing their [ATP] by 0.13 ± 0.04 mM (*n* = 23, *N* = 3) within 2 min (Figure 4). After 5 min, the initial increase in astrocytic [ATP] had reverted into a decrease by 0.22 ± 0.06 mM. At 10 min of a recurrent activity, [ATP] in astrocytes had decreased by 0.37 ± 0.08 mM as compared with baseline (Figure 4). After bicuculline was washed out and standard extracellular Mg^2+^ re-installed, astrocytic [ATP] partially recovered, similar to what was observed for neurons (Figure 4).

Finally, we evaluated whether both ATeam variants would report changes in [ATP] consistently. To this end, we compared the astrocytic increase in [ATP] after initiation of a neuronal network activity. First, we evaluated the changes in the FRET ratio between the two sensors. In astrocytes expressing ATeam1.03^YEMK^, the FRET ratio had increased by on average 0.022 ± 0.007 (Figure 4B) after 2 min. Employing the calibration parameters obtained for this sensor resulted in a change in astrocytic [ATP] by 0.13 mM as reported above (Figures 4A,B). When ATeam1.03 was expressed in astrocytes, the same manipulation resulted in a significantly smaller change in the FRET ratio, namely, by 0.011 ± 0.006 (*n* = 21, *N* = 3; Figure 4B), a behavior expected based on its significantly higher apparent *K*_D_. Converting these changes in the FRET ratio into changes in [ATP] on the basis of the *in situ* calibration yielded an increase by 0.11 ± 0.05 mM, which was similar to that reported by ATeam1.03^YEMK^ (Figure 4B).

Overall, our data demonstrate that induction of a recurrent neuronal network activity in organotypic hippocampal tissue slice preparations results in an initial increase in astrocytic [ATP], whereas neuronal [ATP] declines. Moreover, they show that the peak amplitudes of changes in the FRET ratio of ATeam1.03^YEMK^ and ATeam1.03 expressed in astrocytes in response to a neuronal activity are different, as expected from the different *K*_D_ of the two sensors. Converting these changes into changes in [ATP] based on *in situ* calibrations, however, enables a reliable quantification of the latter independent from the sensor used.

## Discussion

This study introduces a procedure for the quantitative determination of changes in [ATP] in astrocytes and neurons of cultured organotypic brain slices with two variants of the ATP-sensitive FRET-based nanosensor ATeam. After cell-type-specific expression of the sensors, cell membranes were permeabilized with β-escin. This enabled their calibration *in situ*, revealing an apparent *K*_D_ of 2.8 mM for ATeam1.03^YEMK^ and of 9.4 mM for ATeam1.03. A linear fit of the calibration curve for each sensor between 1 and 3 mM translated a 10% change in the FRET ratio into a change in [ATP] by 0.57 mM (ATeam1.03^YEMK^) and by 0.93 mM (ATeam1.03), respectively. Using this method, we demonstrate that neurons in organotypic brain slices respond to a disinhibition and recurrent network activity by an immediate decrease in cellular [ATP] amounting to ~0.3 mM after 2 min. In contrast to this, induction of neuronal activity is accompanied by an initial increase in the [ATP] of astrocytes by about 0.12 mM, followed by a slow decline with persisting neuronal burst firing.

### Strategies for Permeabilization of the Cellular Plasma Membrane

The ATP molecule has a radius of about 0.7 nm and a molecular mass of ~0.5 kDa (Milo et al., 2010). Membrane permeabilization for ATP, therefore, requires non-selective pore formation strategies. To achieve this goal, α-toxin, streptolysin, electroporation, and short freezing/thawing rounds have been commonly used to induce the formation of large pores in cell membranes (Schulz, 1990; Bhakdi et al., 1993). However, most of the latter approaches either result in an only transient permeabilization of the plasma membrane or are not suitable for long-term permeabilization of living cells. An alternative for membrane permeabilization is the use of saponins, which are glycosides that generate pores by creating cholesterol complexes (Kuznetsov et al., 2008; Augustin et al., 2011).

In our hands, incubation of the slices with digitonin resulted in an almost complete loss of fluorescence emission, suggesting a loss of the sensor protein from the cells and/or a membrane disintegration. ATeam itself is a much larger molecule than ATP: the ε subunit of the bacterial FoF1-ATP synthase has a molecular weight of 14 kDa (Imamura et al., 2009); and each fluorescent protein of the FRET pair is around 25 kDa and has a diameter of about 2.4 nm (Hink et al., 2000; Kremers et al., 2011). We then employed β-escin, which is a tripertenoid saponin obtained from horse chestnut seed with anti-inflammatory properties (Domanski et al., 2016). It has been used for the permeabilization of smooth muscle and gastric gland cells, as well as for a concentration-dependent pore formation in perforated patch-clamp recordings from myocytes and neurons (Konishi and Watanabe, 1995; Fan and Palade, 1998; Akagi et al., 1999; Sarantopoulos et al., 2004). As opposed to digitonin, the fluorescence of the FRET pair remained well detectable and allowed imaging of intracellular ATP for as long as 1 h after β-escin was washed in.

Upon permeabilization with β-escin, cells were apparently not fully depleted of ATP, which can most likely be attributed to a remaining metabolic activity in intact mitochondria. β-escin is in fact commonly used to study intact mitochondria *in situ*, permeabilizing mostly the plasma membrane, which is rich in cholesterol, but keeping the membrane of organelles like mitochondria intact (Kuznetsov et al., 2008). Applying blockers for glycolysis and mitochondrial respiration indeed caused a further depletion of ATP from the cells. To further promote the full equilibration of extracellular and intracellular ATP, we also applied orthovanadate, which blocks remaining cellular ATP consumption by blocking an ATPase activity (Cantley et al., 1977). Under these conditions, stepwise changes in the [ATP] in the extracellular saline resulted in stepwise changes in the FRET ratio, allowing us to construct full calibration curves for both sensors in the range of 0–10 mM of ATP.

### Properties of ATeam in Astrocytes and Neurons

The dissociation constant (*K*_D_) of ATeam1.03 determined *in vitro* is 3.3 mM at 37°C; this value dropped about 5-fold (to ~0.7 mM) with a decrease in temperature by 10°C (Imamura et al., 2009). The high-affinity variant ATeam1.03^YEMK^ exhibits a *K*_D_ of 1.2 mM at 37°C *in vitro* (Imamura et al., 2009), suggesting that at room temperature the *K*_D_ is close to 0.2 mM. With reported cellular [ATP] of ~2 mM (Fukuda et al., 1983; Ainscow et al., 2002; Mollajew et al., 2013; Rangaraju et al., 2014; Toloe et al., 2014; Pathak et al., 2015), this suggested that at room temperature (20–22°C) ATeam1.03^YEMK^ might already be saturated under baseline conditions. Consequently, these *in vitro* data implied that ATeam1.03^YEMK^ might not be suitable for reliable measurement of changes in [ATP] and in particular might not be able to sense increases [ATP].

As opposed to this, our recent study showed that transient elevation of extracellular K^+^ resulted in a well-detectable transient increase in the FRET ratio of ATeam1.03^YEMK^ in astrocytes at room temperature (Lerchundi et al., 2019a). This result clearly argued against a saturation of the sensor and pointed towards a possible shift in its *K*_D_ to the right in intracellular environments. The *in situ* calibration curves of ATeam1.03^YEMK^ and ATeam1.03 determined in the present study confirm this notion by demonstrating a significant increase in their *K*_D_’s as compared with those reported from cuvette calibrations (Imamura et al., 2009). Both variants showed Michaelis–Menten kinetics with an apparent *K*_D_ of 2.8 mM (ATeam1.03^YEMK^) and 9.4 mM (ATeam1.03).

Another notable difference between the *in vitro* and *in situ* behaviors of the sensor seems to be its sensitivity to temperature. Imamura et al. (2009) described that in cuvette calibrations, the *K*_D_ of ATeam1.03 was increased approximately 5-fold when increasing the temperature by 10°C. This is in contrast to the results obtained in our former study performed in organotypic tissue slices. Here, we found comparable changes in the FRET ratios of ATeam1.03^YEMK^ at 22° and at 35°C upon elevation of extracellular K^+^ (Lerchundi et al., 2019a), suggesting that inside living cells, the sensor is relatively unaffected by temperature perturbations.

An increase in the *K*_D_ of both ATeam and the ratiometric ATP indicator “Queen” when expressed in cells was reported before and was attributed to differences in the buffer conditions (Yaginuma et al., 2014). Likewise, a shift of the apparent *K*_D_ to higher values inside cells is observed with many commonly used chemical ion-sensitive indicators (Negulescu and Machen, 1990; Bassani et al., 1995; Schreiner and Rose, 2012; Meyer et al., 2019). Moreover, the behavior of genetically encoded calcium indicators (GECIs) is dramatically affected by pH, and ionic strength and composition when expressed *in vivo* (Hires et al., 2008; Pérez Koldenkova and Nagai, 2013).

These earlier observations, together with the findings reported here, emphasize the necessity for a calibration of fluorescent sensors inside cells if quantitative measurements of changes in concentrations are desired. When comparing the full calibration curves of ATeam1.03^YEMK^ expressed in neurons with those of astrocytes in organotypic slice culture, we did not detect any meaningful difference. This indicates that within a given preparation and a given experimental environment, the sensors exhibit similar properties.

In the last years, many new tools were developed for the detection of cellular metabolites, and several were designed for measurement of intracellular ATP (Tantama et al., 2012; Barros et al., 2018). The latter include variations of ATeam (Mendelsohn et al., 2018), including a sensor called “GO-ATeam,” which exhibits a red shift in its excitation/emission spectra, allowing simultaneous imaging with UV sensors like Fura-2 (Nakano et al., 2011). Moreover, ratiometric probes such as the ATP-indicator “Queen” or “PercevalHR,” which report the ATP-to-ADP ratio, were introduced (Tantama et al., 2013; Yaginuma et al., 2014). Notably, such sensors can also be directed to specific cellular organelles like mitochondria (Rueda et al., 2015; Suzuki et al., 2018) or to the plasma membrane (Lobas et al., 2019).

Several FRET-based metabolite sensors have been proven to show high bio-compatibility in animal models such as mice (Mächler et al., 2016). In the case of ATeam, binding of ATP to the sensor does not produce a hydrolysis of the molecule (Imamura et al., 2009), and there is no evidence that ATeam exerts a buffering effect, a phenomenon well described for chemical ion indicators of Ca^2+^ (McMahon and Jackson, 2018). ATeam and its mitochondrial variant mito-ATeam were recently employed *in vivo* for imaging of ATP dynamics in cortical neurons and in peripheral axons (Baeza-Lehnert et al., 2019; Van Hameren et al., 2019). In summary, these results, together with the possibility to perform calibrations as demonstrated in the present study, show that ATeam and its variants are suitable tools for the detection of intracellular ATP in sub-cellular compartments, cell cultures, and tissue slices, as well as *in vivo*.

### Differential Metabolic Response of Neurons and Astrocytes to Neuronal Network Activity

Inhibition of GABA_A_ receptors and activation of NMDA receptors by application of bicuculline and reduction of the extracellular Mg^2+^ concentration induce recurrent neuronal network activity in hippocampal brain slices, resulting in network-wide Na^+^ oscillations in neurons (Karus et al., 2015). In acutely isolated tissue slices derived from transgenic mice expressing ATeam1.03^YEMK^ in neurons (Trevisiol et al., 2017), we could recently show that this neuronal bursting activity is accompanied by a decrease in the intracellular [ATP] by 0.1–0.6 mM (Gerkau et al., 2019). The observed decrease in ATP during recurrent network activity is most likely related to the Na^+^ influx and the resulting activation of the NKA, because previous studies, including our own, have found a direct link between Na^+^ elevations and changes in ATP levels in neurons (Chinopoulos et al., 2000; Mollajew et al., 2013; Gerkau et al., 2019).

While induction of network activity caused a reduction in neuronal ATP, the same manipulation resulted in an initial increase in the [ATP] of astrocytes during the first 2 min of exposure. This is of note because astrocytes, like neurons, undergo transient Na^+^ oscillations under these conditions (Karus et al., 2015). While astrocytic Na^+^ oscillations have lower peak amplitudes as compared with neurons, the accompanying transient increases in extracellular K^+^ additionally stimulate astrocytic (but not neuronal) NKA (Karus et al., 2015), suggesting a significant increase in astrocyte ATP consumption. Taken together, these results clearly indicate that: (1) astrocytes do increase their ATP production in response to neuronal bursting; and (2) this increase can initially override their ATP consumption, resulting in an overall increase in cellular [ATP].

Earlier work has provided evidence as to the underlying mechanisms of neuro-metabolic coupling. Activity-related increases in extracellular K^+^, which result in a depolarization of astrocytes, activate the electrogenic sodium–bicarbonate–cotransporter NBCe1, inducing a cytosolic alkalization and, as consequence, a stimulation of glycolysis (Bittner et al., 2011; Ruminot et al., 2011; Choi et al., 2012). In line with this, an exposure of astrocytes to a brief period of elevated extracellular K^+^ produces a reversible increase in their cellular ATP level (Fernández-Moncada et al., 2018; Lerchundi et al., 2019b).

Notably, the initial increase in astrocytic [ATP] observed here turned into a decrease with prolonged neuronal bursting activity, indicating that the overproduction of ATP is a transient event only. In addition to transient elevations in extracellular K^+^, recurrent network activity is also accompanied by the repeated release of glutamate from neurons. Initially, glutamate might support the K^+^-induced increase in glycolysis by stimulating the transport of glucose into astrocytes (Bernardinelli et al., 2004). Na^+^-dependent uptake of glutamate by astrocytes, however, results in an activation of the NKA, decreasing astrocytic ATP (Chatton et al., 2000; Magistretti and Chatton, 2005; Langer et al., 2017; Winkler et al., 2017). Apparently, the latter gradually overcomes the K^+^-induced increase in ATP production, thereby resulting in an overall decline of the [ATP] in astrocytes with time.

## Conclusions

We show that permeabilizing agents like β-escin allow the calibration and quantitative determination of changes in cellular [ATP] using genetically encoded biosensors in cultured organotypic brain slices. Importantly, both ATP sensors employed here show a significant decrease in their apparent ATP sensitivity when expressed inside cells as compared with *in vitro* conditions and are thus well suited to detect changes in intracellular [ATP]. ATeam1.03^YEMK^ seems preferable to ATeam1.03 because its *K*_D_ as determined *in situ* (2.7 mM) is close to cellular baseline [ATP]. This results in larger changes in the FRET ratio in response to the same perturbation as compared with ATeam1.03, which exhibits an apparent *K*_D_ of >9 mM.

Finally, the protocol described here has the potential to be employed in acutely isolated tissue slices as well. It may thus be applicable to transgenic animals expressing the sensor (Trevisiol et al., 2017) or after sensor expression following injection of viral vectors into the brain. In addition, our methodological approach may be employed for the calibration of other genetically encoded biosensors sensitive to lactate, pyruvate, or glucose (Tantama et al., 2012; Barros et al., 2018).

## Data Availability Statement

All experimental datasets supporting the conclusions of this article will be made available by the authors, without undue reservation, to any qualified researcher.

## Ethics Statement

The experiments performed in this study were communicated, reviewed, and approved by the Animal Welfare Office at the Animal Care and Use Facility of the Heinrich Heine University Düsseldorf.

## Author Contributions

RL performed the experiments, data analysis and interpretation, and manuscript writing. NH performed the preparation and maintenance of cultured organotypic slices, 3-D reconstructions, and documentation and gave the approval of the final version of the manuscript. CR acquired the necessary funding, designed and conceptualized the study, and performed data interpretation and manuscript writing.

## Conflict of Interest

The authors declare that the research was conducted in the absence of any commercial or financial relationships that could be construed as a potential conflict of interest.
